# Experiences and long-term repercussions of perinatal grief in women after perinatal bereavement: a meta-ethnography

**DOI:** 10.3389/fpsyt.2025.1661483

**Published:** 2025-12-17

**Authors:** Beatriz Volpin Gomes Beato, Giovanna Cristina Machado-Kayzuka, Rhyquelle Rhibna Neris, Elana Payne, Willyane de Andrade Alvarenga, Ana Carolina Andrade Biaggi Leite, Fernanda Machado Silva-Rodrigues, Naiara Barros Polita, Francine deMontigny, Sergio A. Silverio, Lucila Castanheira Nascimento

**Affiliations:** 1 Escola de Enfermagem de Riberão Preto, Universidade de São Paulo, São, Paulo, Brazil; 2 Department of Women & Children’s Health, School of Life Course & Population Sciences, King’s College London, London, United Kingdom; 3 Department of Psychological Medicine, Institute of Psychiatry, Psychology & Neuroscience, King’s College London, London, United Kingdom; 4 Department of Psychology, Institute of Population Health, University of Liverpool, Liverpool, United Kingdom; 5 Enfermagem, Centro Universitário Santo Agostinho, Teresina, Brazil; 6 Departamento de Ciencias de la Salud, Universidad Pública de Navarra, Pamplona, Spain; 7 Centro de Ciências da Saúde, Universidade Estadual de Londrina, Londrina, Brazil; 8 Centre d’Études et de Recherche en Intervention Familiale, Université du Québec en Outaouais, Gatineau, QC, Canada

**Keywords:** perinatal grief, bereaved mothers, pregnancy loss, perinatal death, systematic review, qualitative evidence synthesis, meta-ethnography

## Abstract

**Introduction:**

Perinatal bereavement can profoundly disrupt maternal identity and is often accompanied by longer-term emotional suffering. Whilst immediate grief responses have been studied, less is known about how this experience evolves over time. This meta-ethnography aimed to synthesize qualitative evidence on the long-term experiences and repercussions of perinatal grief in women after a pregnancy loss.

**Methods:**

A systematic review of six databases was conducted. Primary qualitative studies were included if they addressed experiences occurring at least one year after a perinatal bereavement. A total of 2,253 records were screened, and 18 studies met the inclusion criteria. Data quality was assessed, and the data were subjected to an analytic synthesis using meta-ethnography.

**Results:**

Three themes and six sub-themes were identified, revealing perinatal grief as a prolonged and transformative experience. Women reported emotional pain, identity disruption, and social silencing. In contrast, empathic care, sustained support, and social validation helped them reconstruct their identities. In line with meta-ethnographic approaches, a theory was developed: *“The quietest of births cause the loudest anguish: Whilst some bereaved mothers walk a solitary path, those with broader support networks are more empowered, but both experience an intense change to The Self.”*.

**Discussion:**

These findings show grief is shaped not only by the loss itself but also by how it is acknowledged or silenced by healthcare systems and society. Gaps were identified regarding long-term grief during times of health system uncertainty and in cases of fetal malformation, revealing the need for further research and policy development.

**Conclusion:**

Supportive and continuous care between lost and future pregnancies is essential to alleviate suffering and promote identity reconstruction among bereaved mothers facing long-term perinatal grief.

## 1 Introduction

Perinatal grief is a distressing experience. It usually begins immediately after a perinatal bereavement caused by an early pregnancy loss (e.g. miscarriage, ectopic pregnancy, molar pregnancy, pregnancy of unknown location, or chemical pregnancy); a later perinatal death (e.g., stillbirth, neonatal death, or early infant death); or after a termination of pregnancy (e.g. early elective abortion, or termination of pregnancy due to fetal anomaly) (1). Perinatal grief involves intense emotions such as sadness, guilt, and suffering, affecting not only the parents but also family dynamics, social relationships, and spiritual well-being (2, 3). Additionally, it can impact future pregnancies, marital relationships, and bonds with other family members (4). In 2025, perinatal mortality remains a major global public health issue, with 2.3 million neonatal deaths occurring within the first month of life, approximately 6,300 per day, and about 1.9 million stillbirths worldwide, many of which could have been prevented with adequate care during pregnancy and childbirth (5). It is also estimated that around 23 million miscarriages occur annually, corresponding to a 15% risk among recognized pregnancies (6). Women in Sub-Saharan Africa and South Asia are the most affected, accounting for 81% of global stillbirths in 2023, 50% in Sub-Saharan Africa, and 31% in South Asia (7).

As a complex and multi-dimensional phenomenon, perinatal grief is universal, manifests after the loss of a baby during the perinatal period (8). However, the lack of a precise conceptual definition in scientific literature hinders its practical application and raises questions about its boundaries in relation to other types of grief (1). Despite growing recognition of the importance of understanding this specific form of grief, which is often neglected by families, healthcare professionals, and society (4, 8). It is an often-invisible experience, and socially invalidated, in cultures that minimize maternal suffering, frequently justifying the loss with the possibility of a future pregnancy (9, 10). Given its wide-ranging and lasting impacts, it is essential that healthcare services and support networks provide adequate assistance to bereaved parents and family members (9).

The emotional pain of perinatal bereavement is profound, and complicated grief occurs frequently amongst women who experience the death of a child (11). The rupture of a deeply meaningful bond contributes to the development of complicated grief, recognized as a health issue which affects the individual, their family, and the community (12, 13). This condition is characterized by deep, persistent, disturbing, and debilitating suffering (13). The loss of the bond, especially due to perinatal death, is a painful and traumatic event which can negatively impact mothers’ lives, abruptly interrupting their hopes and family plans (2). This can result in adverse health implications for these family members, including affective disorders, family disintegration (4), anxiety (14), depression, and even post-traumatic stress disorder (15).

These challenges reinforce the need to better understand the long-term repercussions of perinatal bereavement, which extend beyond the immediate aftermath of death and often remain unaddressed over time. Despite existing reviews, a gap persists in understanding the long-term repercussions of this loss, including accessibility issues and the enduring experiences of bereaved women. ‘Long term’ in this context refers to *“period extending over several years into the future (as opposed to the immediate future)”* (16). In this study, this period is more than twelve months. The Dual Process Model of Coping with Bereavement, proposed by Stroebe and Schut (1999), can support the conceptual understanding of perinatal grief and its long-term repercussions (23). This model conceptualizes grief as a dynamic and oscillating process in which individuals alternate between loss-oriented coping, focused on the pain, memories, and emotional connection with the deceased baby, and restoration-oriented coping, which involves engagement in activities and adaptations that help rebuild life and identity after the loss. The Dual Process Model provides a valuable framework for interpreting the complexity and non-linear nature of perinatal bereavement, particularly over the long term, as women often experience recurrent cycles of confrontation with loss and adaptation efforts throughout their lives (23).

The need for this review is rooted in the absence of interpretative syntheses that bring together and analyze qualitative studies focused on the long-term experiences and repercussions of perinatal grief. Furthermore, no meta-ethnography has been identified to date that addresses this specific focus. Thus, this review becomes essential to advance knowledge and improve the quality of care and support offered to women experiencing perinatal grief. It aims to answer the following question: *“What is the qualitative evidence for the long-term experiences and repercussions of perinatal grief from the perspective of women who have experienced a perinatal bereavement?”*.

We employ a meta-ethnographic approach, which is an interpretive and inductive method that enables the analytic-synthesis of interpretations from different qualitative studies. It generates new understandings which go beyond individual findings, thus rendering more than a qualitative evidence synthesis, but an analytical advancement to the field of study as well (17, 18). This review can contribute to the integration of its results into clinical practice and decision-making processes, including the development of guidelines and the formulation of public policies more attuned to the needs of this population. The findings of this review may also help nurses, midwives, obstetricians, gynecologists, and other clinicians working in this clinical space to adapt their care to this population and implement effective interventions that promote holistic care, making this experience less difficult for bereaved women.

## 2 Method: the systematic review

### 2.1 Design

This systematic review of qualitative studies was registered on the PROSPERO platform (International Prospective Register of Systematic Reviews) under the number CRD42024596532.

**Figure 1:**
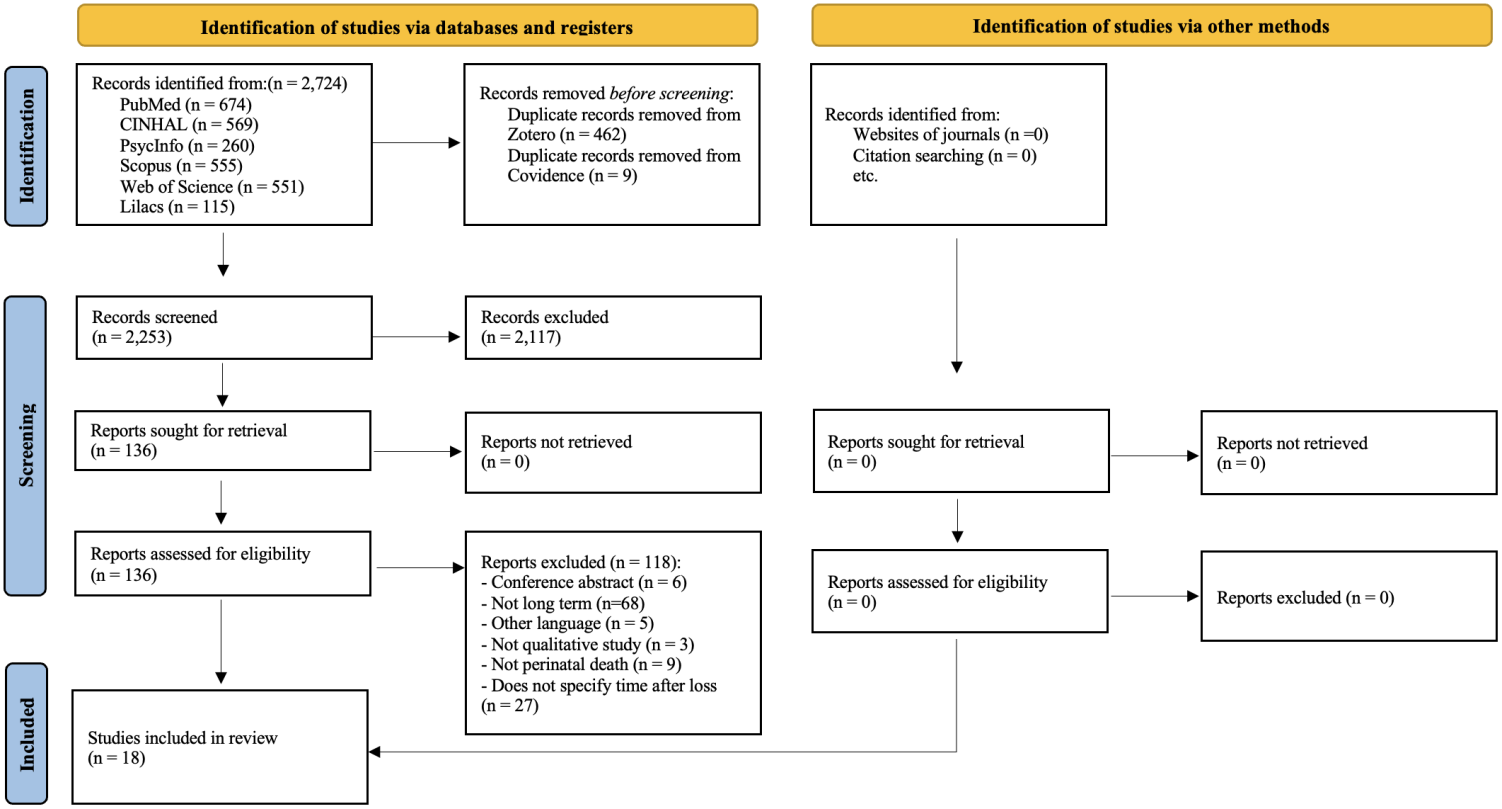
PRISMA flowchart (Page et al., 2021) of the search process and selection of the studies for the metasynthesis.

The PRISMA flowchart (19) was used to report the study selection process, detailed in **Figure 1**. The literature search initially identified 2,724 articles, from which 462 duplicates were removed using Zotero^®^, and nine additional duplicates were excluded via Covidence^®^, totaling 2,253 unique records. After screening titles and abstracts, 2,117 articles were excluded. A total of 136 studies were assessed in full text by two independent reviewers, leading to the final inclusion of 18 studies. Reasons for exclusion at each stage were documented and are presented in the PRISMA flow diagram (**Figure 1**).

This meta-ethnographic review followed a seven step approach (17): 1) Getting started, with the selection of the topic of interest; 2) Deciding what is relevant to the initial interest, with the selection of studies to be included in the review; 3) Reading the studies, with the interpretation of evidence from the included studies; 4) Determining how the studies are related, by comparing recurring themes identified in the included studies; 5) Translating the studies into one another, by comparing and translating the central concepts of one study into the terms and concepts of the others, while respecting the original meanings; 6) Synthesizing translations, by integrating the concepts and generating a new interpretation of the data from the primary studies; and 7) Expressing the synthesis, through the presentation of the results of the meta-ethnography.

To ensure transparency and reporting quality, this meta-ethnography followed the eMERGe recommendations for interpretive syntheses of qualitative evidence. The final phase included three main steps: (1) summarizing the findings, (2) analyzing strengths, limitations, and reflexivity, and (3) developing recommendations and conclusions (18).

### 2.2 Search strategy

The SPIDER tool (Sample, Phenomenon of Interest, Design, Evaluation, Research type) was used to construct the search strategy. The search was structured using the Boolean operators AND/OR to link descriptors and keywords related to the research question. Preliminary searches were conducted to identify literature relevant to the review question and to extract commonly used search terms in studies related to women, perinatal grief, experience, and qualitative research. An example of the preliminary search strategy carried out in PubMed is presented in **Supplementary Table S1**.

Systematic searches were conducted in the following databases: PubMed, CINAHL, PsycINFO, Scopus, LILACS, and Web of Science. To identify articles which were not retrieved through database searches, a manual search was also performed in the reference lists of the included studies and in the references of other literature reviews relevant to the objective of our study.

The search included articles published in Portuguese, English, Spanish, and French, based on the researchers’ language proficiency, regardless of the initial publication date, and included studies published up to 2024.

### 2.3 Eligibility criteria

The inclusion and exclusion criteria are detailed in **Table 1**.

**Table 1:** Eligibility criteria for screening.

Domain	Inclusion criteria	Exclusion criteria
Sample	Women who experienced: – Spontaneous miscarriage (before 22 weeks or <500g) – Stillbirth (after 22 weeks or >500g) – Neonatal death (within 28 days of life)	Studies reporting on women’s experiences only from the perspective of family members, healthcare professionals, or friends.
Phenomenon of Interest	Experience of maternal perinatal grief	Studies reporting maternal losses due to induced abortion
Design, Evaluation, and Research Type	All types of qualitative research – including ethnography, phenomenology, qualitative inquiry, action research, discourse analysis, and grounded theory.	Theses, dissertations, books, reviews, book chapters, and abstracts published in conference proceedings; and studies using mixed methods.
Timeframe	Studies addressing the immediate and long-term repercussions of perinatal grief	Articles portraying only the immediate repercussions of perinatal grief

Perinatal grief has previously been conceptualized as the emotional experience of parents following the loss of a baby, whether due to miscarriage, stillbirth, neonatal death, or elective termination due to fetal malformations (1). The experience of grief may vary between genders, with differences in intensity and duration. Perinatal grief is influenced by various aspects, such as the context in which the loss occurs, individual characteristics, and cultural, religious, and traditional factors. Although there is no specific time frame for this type of grief, supportive practices such as creating memories, naming the baby, holding the baby, and conducting a funeral can help mitigate the intensity and duration of suffering (1, 59).

Definitions of pregnancy loss and perinatal deaths vary globally, which adds complexity to comparing experiences at the international level. For example, the World Health Organization defines stillbirth as the birth of a baby without signs of life at ≥28 weeks of gestation (20); however, according to the 11^th^ edition of the International Classification of Diseases (ICD-11), a baby is considered stillborn when they die within 22 completed weeks of gestation or more (20, 21); whilst in the United Kingdom, baby loss from the 24^th^ week onward is considered a stillbirth (22). There is therefore a blurred line between how different countries determine a miscarriage from a stillbirth, meaning for purposes of international comparison and to broaden understanding of perinatal grief, miscarriage will also be included in this review.

### 2.4 Study selection

The Zotero^®^ reference manager was used to remove duplicate records. The online software Covidence^®^ was used for the initial screening of studies, which was conducted independently by two reviewers (BVGB, GMK) based on the information provided in the titles and abstracts. Subsequently, the same two reviewers assessed the full texts of the selected articles. Any discrepancies were resolved through discussion, and, when necessary, a third reviewer was consulted (LCN).

### 2.5 Quality assessment and confidence in the evidence

The quality of the studies was assessed according to the JBI Critical Appraisal Checklist for Qualitative Research (24). However, considering the nature and purpose of this type of study, quality assessment was not used as a criterion for excluding articles. Two reviewers conducted this process independently (BVGB, GMK), with a third reviewer consulted in case of disagreement (LCN).

The GRADE-CERQual approach (Confidence in the Evidence from Reviews of Qualitative Research) was used to assess confidence in the findings. Confidence is defined as the evaluation of the extent to which a review finding reasonably represents the phenomenon of interest. This confidence assessment is based on four components: (1) Methodological limitations of the qualitative study contributing to a review finding; (2) Coherence, referring to how well the review finding is supported by data from the primary studies; (3) Quantity and adequacy of data, with a degree of richness that supports the review findings; and (4) Relevance, meaning the applicability of the primary data to the review’s research question (25).

### 2.6 Data extraction and synthesis

Two reviewers (BVGB, GMK) independently extracted data from the included articles, with data verification performed by a third experienced reviewer (SAS). Initially, due to the objective of this review, the following data were extracted: Study (country, author, and year), Objectives, Participants (sample size, data source/sampling, number of losses, gestational or neonatal age, diagnosis causing the loss, time between loss and interview, focus on dimension of the loss, private health insurance), Methods (study design, research type), Outcome measures, Study quality, and results that objectively present an experience.

The analysis of the extracted data was conducted through meta-ethnography (17). In the first phase, the focus of the review was identified: understanding experiences related to perinatal bereavement. In the second phase, inclusion and exclusion criteria for studies were defined, as well as procedures for assessing the methodological quality of the selected studies (26). The third phase consisted of detailed and repeated reading of the studies to identify first- and second-order constructs present in each study. In the fourth phase, the studies were compared to determine how they relate to each other (17). From this comparison, recurring and shared concepts were identified, also analyzing the relationships between metaphors and central themes to highlight the most relevant ones (26). Emerging themes were then grouped into meaningful analytical categories (27). This process was iterative and involved discussions among the review team to refine interpretations and ensure the original context of each study (26). In the fifth phase, concepts and themes from one study were “translated” into the context of another study, allowing for identification of similarities or differences. This process enables organizing convergences and divergences among concepts into broader categories, culminating in the construction of third-order interpretations (27). The sixth phase was divided into three steps: reciprocal synthesis, refutational synthesis, and line-of-argument synthesis (26). In reciprocal synthesis, common themes were integrated into first- and second-order constructs, generating new concepts called third-order constructs (28). Refutational synthesis focused on identifying and comparing contradictions between studies, a process repeated until the third-order constructs were formed (26). Based on these constructs, a line-of-argument synthesis was developed, supported by the continuous comparison of concepts and the construction of a theory that organizes similarities and differences among the studies (17). In the final phase, the synthesis was expressed through a conceptual model or interpretative narrative, clearly communicating the higher-level metaphors or concepts derived from the analysis. This synthesis not only integrates study findings but also contributes to theoretical development, generating insights that can guide future research and practice (17, 18).

## 3 Results: qualitative evidence synthesis

### 3.1 Characteristics of the included studies

Eighteen qualitative studies published between 2002 and 2024 were included in the review. These studies were conducted in Brazil (n=4), Australia (n=3), the United Kingdom (n=3), Canada (n=2), Israel (n=2), Italy (n=1), Sweden (n=1), France (n=1), and Japan (n=1).

In total, the 18 studies included 242 women who experienced different types of perinatal bereavement. Seven studies addressed miscarriage, 13 investigated stillbirth, and ten analyzed grieving experiences after neonatal death. The average time since the loss was 5.31 years, excluding studies that did not report these data precisely. The focus on perinatal bereavement varied across the analyzed studies. Half addressed support networks and social support (29–36). Maternal identity and motherhood were explored in seven articles (29, 32, 37–41). Just five articles reported on grief and psychological impacts (32, 37, 40–42), or health-related aspects (34, 35, 43–45). Psychosocial aspects were addressed in just four articles (29, 33, 34, 42). Return to work was the focus of only two articles (34, 46). Finally, the experience of a subsequent pregnancy after loss was discussed again, just two articles (33, 37). The full characterization of the 18 included studies is presented in **Table 2**.

**Table 2:** Summary of included studies.

First author, year, and country of reference	Objective	Participants (n) and type of perinatal loss	Time after loss	Main results and conclusion
Borges, 2024 Brazil (34)	To understand the experience of returning to work for women who experienced a spontaneous abortion	3 women - Losses at 23 weeks’ gestation	2 years	The study explored pregnancy and pregnancy loss, revealing that participants faced complications that affected their grief processing. They reported a lack of empathy from some healthcare professionals during the loss notification and clinical interventions. However, positive experiences were noted with primary healthcare providers, who offered consistent support. Participants expressed feelings of emptiness after the loss. The findings suggest that healthcare environments lacked the necessary tools and professional skills to effectively manage grief.
Rossen, 2023 Canada (38)	To investigate how women construct a sense of maternal identity after the loss of a baby	10 women who experienced perinatal loss, including neonatal death, stillbirth, and miscarriage	More than 5 years (mean = 5)	The study explores how mothers navigate identity and grief after pregnancy or infant loss. Despite emotional pain and feelings of invalidation, participants found meaning, gratitude, and personal growth through their experiences. They transformed loss into a way to honor their child and support others. The research aims to break the silence, stigma, and confusion surrounding such losses. It also seeks to inform and support clinicians, researchers, and families in these situations.
Teodózio, 2022 Brazil (41)	To identify and comprehend the perceptions and feelings of mothers about their subsequent pregnancy and baby after a pregnancy loss	4 women - Losses from 16 to 40 weeks, plus one neonatal death (2 days old)	2,5–4 years (mean =3,25)	The study identified two main themes: Maternal Feelings About Pregnancy and Maternal Perceptions and Feelings About the Baby After Birth. The first theme revealed fear and concern about another pregnancy loss, ambivalence about the desire to have the baby, anxiety regarding delivery, and a desire to conceal the pregnancy from family. The second theme highlighted the impact of previous loss on perceptions and emotions about the baby, including idealization of the baby, persistent fear of losing the baby, and evidence of substitution, such as choosing similar names and reusing items from the deceased baby.
Wheeler, 2022 UK (30)	To explore the subjective experiences of women who had lived through a perinatal loss and a subsequent pregnancy, and particularly what support made a meaningful difference to their experiences	41 women – Gestational losses before 12 weeks, between 12 and 23 weeks, between 24 and 42 weeks, and four neonatal deaths	1–5 years (mean = 3)	The need for developing a common language seemed to be an important part of breaking the silence and taboos around baby loss. However, these participants’ experiences show the importance of this language being developed by rather than for bereaved parents, as well as the need for healthcare professionals to be aware of the power of the labels they use, and to be inclusive of the subjective experiences of those who they care for. Empathic care requires sensitivity to the way in which terminology can both validate and dismiss distress experienced by bereaved mothers.
Lopes, 2021 Brazil (42)	To understand maternal feelings towards perinatal death	23 women with pregnancy loss at 22 gestational weeks or early neonatal death (0–6 days of life)	1 year	With this study, it was possible to understand the maternal feelings facing perinatal death, such as shock, sadness, guilt, pain, and fear, highlighting the utmost importance of creating support networks capable of assisting these mothers and helping in this difficult process.
Testoni, 2020 Italy (29)	To identify social strategies that psychologists and social workers can use to support specific forms of mourning	15 women - Participants experienced perinatal loss between the fifth and the ninth month of pregnancy	6.45 years	The study considered the relationship between perinatal bereavement and CBs, highlighting the crucial role played by both the mourner’s husbands/partners and by their social network in helping the mothers to begin a healthy mourning process. Supports made a significant difference since the participants who could not count on such a support faced isolation, stigmatization, extreme sorrow, and ultimately unresolved grief, with the strong presence of CBs.
Devincenzi 2019 Brazil (35)	To understand the experiences of women who experienced neonatal deaths in a vulnerability region of the Municipality of Santos, São Paulo, Brazil, examining them from the perspective of vulnerability of women and the comprehensiveness of health care involved	8 women - Neonatal death	1 year	These births were considered avoidable with adequate care, but failures in reception and management of complications during labor worsened outcomes, including neonatal deaths. In the postpartum period, health care was limited. Only two cases had a postpartum consultation registered and carried out in a timely manner, while the lack of records in the private sector prevented verification of care for some women. Severe cases highlight critical failures in monitoring, resulting in another fetal death and risk of death for the mother. In addition to the clinical consequences, neonatal deaths generated profound emotional and social impacts. Some women faced depression, financial and health difficulties, while cases of intimate partner violence remained to health services.
Due, 2018 Australia (44)	To explore heterosexual women’s experiences of the healthcare system in Australia and the care they received after a pregnancy loss	15 who experienced perinatal loss between 6 and 40 weeks of gestation (mean = 18 weeks)	2–47 years (mean = 24.5)	This study highlights both negative and positive experiences of women after pregnancy loss. Negative aspects included unclear, clinical language, inadequate hospital environments, lack of emotional support, and insufficient follow-up care. Many women felt isolated and sought private psychological help. Positive experiences involved compassionate healthcare staff, empathetic communication, and individualized care such as home visits. Holistic support addressing both physical and emotional needs was especially valued.
Jordan, 2018 UK (40)	To explore the experiences of mothers bereaved after loss of a twin from a multiple birth pregnancy, focusing on the dual challenges of parenting and grieving	18 women – Gestational loss (gestational age not specified)	1–20 years (mean =10.5)	This study explores the experiences of mothers who lost one twin, focusing on three key themes. First, mothers struggled to talk about their deceased child, adjusting their responses based on emotions and the audience. Second, they navigated honoring the lost twin while celebrating the surviving one, often using personalized coping strategies. Third, mothers experienced a changing sense of self, grieving the loss of their anticipated “twin parent” identity and feeling frustration over others’ lack of acknowledgment. The study highlights the emotional complexity of balancing grief and joy after twin loss.
Meredith, 2017 Australia (45)	To understand the experience of pregnancy and birth for mothers in a pregnancy following perinatal loss, and to understand their experience of the specialized Pregnancy After Loss Clinic (PALC) provided at the Mater Mothers’ Hospital in Brisbane, Australia.	10 women - Stillbirth to 24 days of life	1–7 years (mean = 4)	This study identified seven themes related to experiences with a Pregnancy After Loss Clinic (PALC). Mothers described PALC as compassionate and essential for emotional well-being, also noting benefits for partners despite participation barriers like work schedules and limited awareness. Inclusion of children and extended family in PALC support was viewed positively. Pregnancy after loss brought unique challenges, such as increased anxiety, grief, and fear of bonding, which PALC staff addressed with empathy. Coping strategies included social support, short-term goals, and memory boxes. Many women felt isolated due to a lack of understanding from family and friends. The impact of loss extended to partners, older children, and relatives, indicating the need for broader, family-centered support.
Golan, 2016 Israel (39)	To examine the meaning that women who experience stillbirth ascribe to their loss in general and to the lost figure in particular	10 women - From 23 weeks	1–9 years (mean =5)	This study describes stillbirth (SB) as an ambiguous loss, with the lost figure seen variably as a baby, fetus, or spiritual presence. Women struggled internally with the meaning of the loss and their maternal identity. Externally, healthcare and societal invalidation intensified confusion and emotional conflict. Mixed messages from professionals and social minimization led some to question or downplay their experience. These internal and external ambiguities made it difficult to find meaning and closure.
Üstündağ-Budak, 2015 UK (37)	To focus on the meaning of the stillbirth experience to women and its influence on the subsequent pregnancy and subsequent parenting from the mothers’ own perspectives	6 women who experienced perinatal loss between 25 and 41 weeks of gestation	15 to 20 months (mean = 17,5 months)	Women’s accounts revealed that the experience of stillbirth is a process where women re-visit the experience and reflect their experiences throughout other life events such as the arrival of a new baby. The experience of stillbirth appears to influence the relationship with the subsequent infant and parenting.
Gagnon, 2013 Canada (46)	To describe and analyze the return-to-work experiences of employees experiencing perinatal grief	9 women whose infants were born at 21 to 40 weeks of gestation and died between 12 hours and 25 days of life	2–33 years (mean = 17.5 years)	This study examines organizational practices following perinatal loss, focusing on leave, return-to-work, task adjustments, and emotional support. Most participants reported insufficient leave and pressure to return quickly, sometimes needing medical proof. Progressive return options were rare but helpful when offered. Task adjustments were uncommon, causing stress, especially in performance-based roles. Emotional support was seen as vital but inconsistently available.
Vasilescu, 2013 French (36)	To describe the resentment of these parents at the tenth birthday of their twins in the neonatal period; the emotional impact of this date; the way in which they are not available to invest in their child living; and, finally, how nursing teams can help parents cope with the difficulties specific to this situation	15 mothers – Gestational age: 27.8 ± 4.4 weeks; lifespan: 10 ± 10.9 days	2–4 years (mean = 3)	Between the loss of a single child and the loss of a twin, the issue is not limited to the difference in the intensity of the parents’ grief, but to the coexistence of very strong emotions: joy and sadness, hope and despair, investment and detachment. During hospitalization and the subsequent follow-up, parents are often challenged to deal with these contradictory feelings. It can be expected that, over the course of the process, parents will go through a journey that allows them to integrate the loss, invest in the living child, and find ways to keep the representations of the children as distinct as possible, in order to preserve each child’s place in the family history. This delicate balance between mourning and living in the present requires constant adaptation, where parents seek ways to honor the memory of the deceased child while caring for and protecting the surviving child.
Yamazaki, 2010 Japan (33)	To describe the meaning of Intrauterine Fetal Death (IUFD) in the lives of Japanese women in a local community, by interviewing those who experienced IUFD after 28 weeks of gestation, in chronological order from the time they were told of the fetal death to the present day	17 women – Losses after 28 or more weeks’ gestation	1–6 years post bereavement (mean = 3,5)	The 17 women who had experienced intrauterine fetal death raised their dead children through “the development process of becoming a parent,” as demonstrated in this study; in addition, through “the grieving process after the loss of a child,” they went through a year-long process of grieving. During that period, these women came to terms with their partners and family members, and they found child-rearing friends to help raise the dead child and were living with the dead child as if the child were a family member.
Gerber-Epstein 2009 Israel (31)	To observe the subjective experience of the woman who has miscarried a first and early pregnancy	19 women – Gestational losses between the 6th and 15th week	1–4 years since loss (mean = 2,5)	This study examines women’s sources of support, coping strategies, and recommendations after miscarriage. Support came mainly from partners, family, and women with similar experiences, though responses varied. Returning to daily life was emotionally difficult, with grief often described as persistent and overwhelming. Work was a distraction for some, but others struggled with societal indifference. Women recommended empathetic healthcare, access to counseling, and freedom to grieve at their own pace.
St John, 2006 Australia (32)	To explore how women experience prenatal loss, the meanings it has for them and how they dealt with the grief and despair	3 women – Losses ranging from miscarriage to neonatal death (not specified)	Not described; all had ≥2 miscarriages and 2 living children	This study explores the emotional impact of prenatal loss, marked by grief, guilt, and isolation. Women felt excluded from maternal identity due to the lack of visible signs of motherhood and limited societal recognition. Support from family, friends, and healthcare professionals was often inadequate, deepening their sense of loneliness. Self-help groups provided comfort, but subsequent pregnancies triggered fear and emotional detachment. Many avoided antenatal classes, highlighting gaps in traditional care for those who experienced pregnancy loss.
Lundqvist, 2002 Sweden (43)	To focus further on and illuminate mothers’ lived experiences of the professional care they had received while facing the threat and the reality of losing their baby	16 participants - Newborn (from 15 minutes to two weeks)	2 years	The study identified two main themes: empowerment and powerlessness, both experienced by all mothers. Empowerment arose from empathetic, supportive care and clear communication from healthcare professionals. Individualized attention and involvement in decision-making provided comfort and confidence. Powerlessness stemmed from poor communication, emotional disconnection, and feeling disrespected by staff. Most mothers felt unsupported, while a few found strength in challenging hospital policies.

### 3.2 Quality of the included studies

Overall, the studies demonstrated adequate congruence between the objectives, methodological approaches, and data collection and analysis methods. However, only one study fully met all ten criteria established by the appraisal tool (34). A recurring issue was the absence of reflexivity regarding the researcher’s positionality and its influence on the investigative process. Specifically, 14 studies did not provide a clear statement regarding the researcher’s cultural or theoretical positioning (29–36, 39–43, 46), and 14 made no mention of the researcher’s influence on the research process (29–35, 39–44, 46). The detailed results of the quality appraisal are presented in **Supplementary Table S2**. No study was excluded based on this assessment. The level of confidence in each finding was assessed using the GRADE-CERQual approach, as shown in **Supplementary Table S3**.

## 4 Results: meta-ethnography

To more clearly represent the developed theory, we present below the themes and sub-themes identified in the analytic-synthesis: 1. Between Care Failures and Sensitive Practices; 2. A Patchwork of Support and a Network of Care; 3. A Grieving Mother Is Still a Mother. Excerpts from each study are presented in **Tables 3**–**5**.

**Table 3:** Example extracted data from the studies relating to the theme of ‘between care failures and sensitive practices’ and its sub-themes.

Sub-themes	First-order constructs: example quotations from study participants	Second-order constructs: authors’ interpretations of original findings
Weaknesses of Care: Lack of Support During the Loss Journey	“… My impression was that ‘you went through a procedure and now you can go home.’ There was no emotion attached to it. [ … ] At no point after my miscarriages did I feel that any healthcare professional or team was overly understanding or empathetic toward me.” (Jenny) (44).	Negative experiences (Confusing and inappropriate language and communication/The hospital environment/Lack of emotional care/Lack of follow-up care) (44).
	“The doctor even said to me: ‘Oh, better now than at nine months, right?’” (Cristal) (34).	Speech from healthcare professional perceived as dismissive (34). Aspects related to pregnancy and pregnancy loss/Regarding the experience in healthcare services (34).
Sensitive Practices: Institutional Support in Perinatal Grief Care	“I got along really well with my obstetrician, who was my age and absolutely lovely.” (Nancy) (29).	Generational differences among professionals; individualized attention (29). Relationships Are Crucial Support Systems (29).
	“The psychotherapist was a lifesaver for me. She helped me a lot during the time of the loss.” (29)	Psychotherapy offered by hospital as a lifesaving support (29). Relationships Are Crucial Support Systems (29).

**Table 4:** Example extracted data from the studies relating to the theme of ‘a patchwork of support and a network of care’ and its sub-themes.

Sub-themes	First-order constructs: example quotations from study participants	Second-order constructs: authors’ interpretations of original findings
Shaped by Relationships: The Duality of Loss	“There were some with empathy, others clueless, others ‘get pregnant soon’, cold.” — Pérola (34).	Insensitive comments from colleagues. Work life and pregnancy loss/How miscarriage was treated in the workplace (34).
	“My friends rarely showed up or text me, so they were little help sadly.” — Isabel (37).	Questioned self and others (37). Isolation in grief due to friends’ absence (37).
Coping with Perinatal Grief: The Role of Social and Emotional Support	Someone would say she understood how I felt. I don’t know if she’d been through the same sort of thing or not, but she understands. It got on my nerves so badly! What do I care if you understand? So what if you understand, does it help me to feel better? No! It’s so annoying! Patronizing! Frustrating! (Zohar, p. 4, line 90) (31).	Sources of support (31). The women described the inability of the support factors, however, central and important, to provide comfort and aid or, alternatively, to provide support for some length of time (an exclusive experience of the woman) (31).
	One mother “focused on her [twin and younger] children” after the death to “avoid thinking, grieving her pain.” She declared a state of enthusiasm, impatience and anger, which led her to seek help with her children: she could not “stand them any longer” (36).	Role of caregivers during hospitalization/Support for parenting during the hospitalization of the living child (36).

**Table 5:** Example extracted data from the studies relating to the Theme of ‘A Bereaved Mother is Still a Mother’ and its sub-themes.

Sub-themes	First-order constructs: example quotations from study participants	Second-order constructs: authors’ interpretations of original findings
From Expectant to Bereaved Mother: Transformations in Maternal Identity	Oh gee petrified — but over the moon, absolutely petrified and not really hopeful … it’s happened this month and I’m pregnant, but then … the over the moon feeling disappears about half an hour after you read the little urine stick … you hold back on any other feelings. (Sally) (32).	Being changed or transformed by loss (32). Loss profoundly transformed the women’s identity; they identified themselves as mothers without children (32).
	“I feel like a mother in the abstract sense of the matter…” (39)	Essence of SB: Prolonged Internal Ambiguity (39). Undefined maternal identity, with questions about whether the experience made them mothers (39).
Re-interpreting Loss after Birth: Coping with Grief and Emotions in a Subsequent Pregnancy	“Every friend of mine who got pregnant was to me—boom and shock! There, she succeeded and there, I didn’t. [ … ] So I avoided them.”*(Tina, p. 9, line 244)* (31)	Fear of not being able to conceive again; subsequent pregnancy surrounded by fear and emotional detachment (31).
	“I summarize this pregnancy as one filled with fear, with a lot of nervousness, a lot of fear (…) like, of it happening again (…) Every moment, like, if she stopped moving, I would get scared, so I would run anywhere, straight to the maternity ward, straight to the clinic.” (41)	Maternal feelings surrounding pregnancy/Constant Fear: Ongoing Concern (41). Maternal Feelings of Fear and Worry. The new pregnancy was marked by fear, anxiety, and ambivalence (41).

### 4.1 Between care failures and sensitive practices

Studies included in this theme revealed that the experience of perinatal bereavement is deeply influenced by the role of health services, fluctuating between abandonment and support, see **Table 3**. On one hand, care failures, insensitive communication, lack of emotional support, and inadequate postpartum follow-up exacerbated women’s suffering (29, 32, 34, 35, 43, 44, 46). On the other hand, sensitive practices such as active listening, psychological support, and professional empathy facilitated grief processing and a sense of being cared for (29, 34, 36, 43–46).

#### 4.1.1 Weaknesses of care: lack of support during the loss journey

The experience of perinatal bereavement was strongly associated with deficiencies in health services and prenatal care, compromising both clinical outcomes and women’s subjective experiences (34, 35). This scenario was marked by institutional failures, inadequate support (44), and a lack of coordination between care levels, all of which intensified maternal suffering in the face of loss (35).

Even when prenatal care began in a timely manner in primary care, the ineffective management of complications such as infections and bleeding led to preterm births, fetal demise, and neonatal deaths (35). These weaknesses highlight the need for active surveillance and proper clinical management, especially when risk signs are present (35). The inadequacy of the physical environment was another recurring factor, such as the placement of grieving women near laboring mothers or newborns, which caused additional distress. Overcrowding, long waiting times, and lack of privacy heightened feelings of neglect and abandonment (44).

Failures in care and labor management also had significant impacts, such as the lack of continuous monitoring, which exposed pregnant women to avoidable risks and resulted in neonatal and maternal deaths (35). The way the loss was discovered and the moments following miscarriage were marked by intense physical and emotional pain. The lack of psychological support from professionals was a frequently reported weakness (34, 44). Although physical care was generally provided, emotional aspects were neglected. Women emphasized that, although healthcare professionals addressed their physical needs, emotional support was lacking (44). This excerpt contributes to the development of the emerging theory by illustrating how the absence of psychological and empathetic care during such an anguishing and painful moment of loss deepens maternal suffering. It reinforces that bereaved mothers’ anguish extends beyond physical recovery, highlighting their need for emotional and psychological support from healthcare professionals. Technical and insensitive terms, such as “retained products” to refer to fetal remains, were experienced as dehumanizing (44). This attitude may reflect a lack of training in breaking bad news or the normalization of loss in the professionals’ routine (44). The reports ranged from experiences of empathetic support and sensitive listening in primary care to others marked by cold and disrespectful speech (34). Situations involving detachment, insensitive comments, or denial of contact with the baby after birth intensified suffering, feeding feelings of helplessness, insecurity, and emotional isolation (43).

In the postpartum period, care for grieving women was limited and unequal. In general, healthcare professionals did not offer follow-up after the loss, which led many women to grieve alone (32). For women in socially and economically vulnerable contexts, the challenges were even greater (35). Only two cases reported timely postpartum consultations, while the lack of records from care provided in private facilities hindered both institutional accountability and continuity of care (35). After hospital discharge, the absence of structured follow-up and specific referrals for emotional support contributed to the feeling of abandonment, with reports of insufficient or nonexistent follow-up during the most critical moments (33, 43). In some cases, care resumed only months after the loss, revealing major shortcomings in mental health care for these women (35).

Finally, returning to work proved to be another space of abandonment. While some organizations demonstrated sensitivity, most did not offer psychological support or recognize the particularities of perinatal grief, thus worsening emotional and professional challenges (46).

#### 4.1.2 Sensitive practices: institutional support in perinatal grief care

In light of the care shortcomings and the fragmentation of health services which intensify the suffering of women experiencing perinatal grief initiatives have emerged aiming to transform this reality through sensitive practices and specialized institutional support. Although the experience of loss is marked by intense pain, studies show that empathetic support from the healthcare team can mitigate this suffering by offering essential assistance during this critical time.

Women’s experiences with healthcare services after perinatal bereavements revealed the coexistence of contrasting care practices: while some reported abandonment, others experienced moments of compassion and sensitivity from professionals, which positively influenced their grieving process and their perception of the quality of care received. Professional support, communication, the hospital environment, and post-event follow-up were central aspects of these women’s experiences (44). Empathetic attitudes from healthcare providers fostered emotional comfort, a sense of empowerment, and support in reconstructing the subjective bond with the baby, contributing to the grieving process (43).

Reports acknowledging institutional support and empathetic care were more frequent in specialized care settings, such as private hospitals or emotional support services (34, 44, 45). The availability of contact with midwives through various channels (phone, messages, or email) was perceived as a continuous support network (45). Small gestures, such as offering a hug or conducting home visits, were described as actions with a significant impact on the grieving experience (45). Cases in which care was delivered holistically, addressing both physical and emotional needs, were valued by women as examples of good care practices (44).

One particularly meaningful memory was the creation of a keepsake collection by the nursing team in honor of the stillborn baby, a gesture considered to hold great symbolic value for the mother (44). Although this high level of support was not shared by all study participants, it illustrates the positive impact of compassionate and sensitive professional interaction. Even outside specialized settings, professionals who showed personal engagement, skilled listening, and physical presence were remembered positively (45). Cases in which care was provided comprehensively, addressing both physical and emotional aspects, were recognized as good care practices (44).

Additionally, psychotherapy groups organized by hospitals were identified as important sources of emotional support (29). Many participants described what they called “surviving through psychological care” during hospitalization, highlighting the importance of this support. Only a small number did not wish to receive this kind of assistance (36). In one study involving the loss of a twin, parental follow-up extended for several months after the death, fostering the bond with both children and allowing emotional expression. Throughout the hospitalization, professionals remained available and attentive to the family’s needs (36). Some participants benefited from psychological support through corporate programs, which, combined with a gradual return to work, enhanced their ability to cope and improved their productivity (46).

### 4.2 A patchwork of support and a network of care

The studies included in this theme contributed to the understanding that perinatal grief is a complex and relational experience, whose intensity and meaning are deeply influenced by the quality of the bonds formed after the loss (30–38, 40, 45, 46). They also address the importance of social support, emphasizing how a lack of recognition and empathy can intensify suffering (29–31, 33, 34, 37, 38, 42, 45, 46).

#### 4.2.1 Shaped by relationships: the duality of loss

The studies highlighted that external support, empathy, and encouragement were elements that could foster resilience, whereas detachment, unmet expectations, and lack of connection contributed to feelings of helplessness, insecurity, and grief. Women faced intense internal emotional struggles, marked by pain, anger, and self-blame (29, 32–34, 36, 40, 45), in a context where perinatal bereavement is often silenced and socially neglected (30, 32, 37, 38, 40). The evidence from included studies reinforces the importance of emotional support in this experience, revealing the duality of loss: it can be softened by care or exacerbated by isolation (32, 45). Perinatal grief is a dense emotional experience that goes beyond the woman’s private suffering and is profoundly shaped by interpersonal relationships and the social context in which it occurs.

Emotions such as guilt, powerlessness, deep sadness, and the effort to maintain emotional balance are mediated by the quality or absence of connections with partners, family members, healthcare professionals, and support networks (29, 32–34, 36, 40, 45). The studies also showed perinatal grief transcending an individual pain, constituting an emotional and symbolic journey marked by rupture, silence, and the ongoing search for meaning in what was conceptualized as ‘motherhood interrupted’ (33, 37, 38).

Suffering is intensified in social contexts that delegitimize gestational loss, especially when family, friends, and healthcare professionals fail to acknowledge the woman’s pain (29, 30, 32, 37, 38, 40). Social stigma labels the woman as *“the mother of the baby who died”*, preventing her from exercising her natural role and deepening her isolation (29, 46). This combination of silence and invalidation can lead to deep frustration and even to questioning the meaning of life itself (30, 37–39). Many describe this experience as *“a silent birth without the child”* (34), which further underscores how perinatal grief is deeply shaped by the quality of relationships established after the event (32).

Despite the intense pain, many participants reported that, over time, they were able to assign new meanings to the loss, experiencing feelings of gratitude and identifying opportunities for personal growth. In this process, social support played an essential role in the grieving process (33), even helping reduce anxiety (32). Support, combined with the symbolic preservation of the baby’s presence, enabled the integration of the grief experience into the continuity of life. The memory of the child, recognized as a meaningful presence within the family, strengthened the reconstruction of meaning and adaptation to the new reality (33). Thus, the journey toward acceptance is marked by emotional ruptures, social silences, and a gradual process of adaptation, in which the re-signification of grief becomes possible (33, 38).

#### 4.2.2 Coping with perinatal grief: the role of social and emotional support

The support provided by partners and social support networks proved to be a key protective factor in coping with perinatal grief. Mothers who received consistent emotional support described healthier and more adaptive grieving processes (29, 31, 35). In contrast, the absence of such support contributed to feelings of loneliness, deep sadness, and persistent suffering, favoring the chronicity of pain and the development of maladaptive coping strategies (29).

The closest support network, especially the partner, was identified as a central element in facing the pain. Many women reported that their partner was their main source of emotional support, highlighting the importance of shared suffering and open dialogue during the grieving process (29). However, difficulties in sharing this experience were also reported, attributed to individual differences in how grief is expressed. Some women stated their husbands avoided conversations about the loss, even though they felt the need to talk about it, which led to frustration and feelings of isolation (29). Social expectations regarding male behavior were pointed out as one of the factors that intensified this disparity, especially due to the pressure for emotional self-control and the early return to work, elements that contribute to the invisibility of paternal grief (33, 37). In some accounts, women said they avoided sharing their feelings because they noticed their husbands were exhausted from a heavy workload (37). Whilst some women expressed their emotions more frequently and verbally, men tended to remain silent, internalize their suffering, or prioritize the role of emotional support for their partners (29, 33). This discrepancy generated feelings of misunderstanding and loneliness (33). Despite these difficulties, some accounts highlighted moments when men were able to express their pain spontaneously, such as a father who cried while holding a relative’s newborn baby, evoking empathy from his partner and revealing the emotional impact of the loss (33).

Broader social support was described ambivalently. Many women reported insensitive or inappropriate attitudes from friends and colleagues, expressed through minimizing remarks or complete withdrawal, thereby intensifying feelings of exclusion (30, 37, 42, 45). A lack of empathy in everyday speech was also noted, with inappropriate comments, pressure to conceive again, and attempts to silence grief, revealing society’s difficulty in dealing with perinatal bereavement (34, 42, 45).

Although some people in their social circles showed empathy, many participants emphasized the discomfort caused by inappropriate approaches. To minimize such awkwardness, some women chose to take the lead in communication, proactively informing colleagues about the loss in order to avoid uncomfortable situations (34). The absence of similar experiences among friends also hindered connection and mutual understanding, making mothers feel disconnected from those relationships (38). In addition, there were reports of people withdrawing because they did not know what to say in the face of loss (30).

Despite the challenges faced, some positive experiences were reported, especially related to the strengthening of family bonds after the loss. In certain cases, the pain of grief served as a catalyst for greater emotional closeness between mother and daughter, or between partners, creating spaces for mutual care and sensitive listening (29, 31).

### 4.3 A bereaved mother is still a mother

The studies in this theme revealed that the experience of motherhood after a perinatal bereavement is marked by a continuous process of emotional and identity reconstruction that does not end with the baby’s death (30–32, 36, 38–40, 42, 43, 45). Moreover, a subsequent pregnancy demands a reinterpretation of motherhood (30–32, 36– 37, 41, 42, 45).

#### 4.3.1 From expectant to bereaved mother: transformations in maternal identity

Perinatal bereavement triggers profound transformations in maternal identity (32, 38, 42). This identity reformulation does not occur linearly and is shaped by internal factors, such as emotional conflicts and ambivalence, and external ones, such as the support received and the social recognition of motherhood (30, 39). In this sense, women began to question whether they could be considered mothers as part of their identity (30, 39).

The absence of the baby, as well as of physical and social markers of motherhood such as childbirth, the postpartum period, and caring for a newborn, led many women to feel excluded from the maternal experience and to question their actual belonging to this role. Some saw themselves as *“abstract”* or *“incomplete”* mothers for not having a living child in their arms (31, 32, 39). This sense of identity displacement was reinforced by insensitive healthcare systems and a culture that minimizes perinatal grief, contributing to women’s isolation and the weakening of their identity (32, 42). This illustrates how perinatal loss profoundly challenges women’s sense of self and motherhood. It reveals how grief extends beyond the loss of the baby to a perceived loss of feminine and maternal identity, reflecting the deep internal transformations that characterize this process. In specific contexts, such as the loss of one of a pair of twins, identity re-definitions became even more complex, with women mourning not only the lost baby, but also the anticipated identity of being a *“mother of twins”* (40).

The way each woman finds her own pace to deal with the experience of identity re-signification is deeply linked to access to empathetic care, the available support network, and the possibility of expressing grief in a legitimate way (30, 42, 45). Emotional support was identified as a central element not only for adapting to perinatal grief but also for rebuilding maternal identity after the loss (30, 42, 45). When present, this support allowed women to recognize themselves as mothers, even in the absence of the baby, integrating the loss into their personal history. On the other hand, the absence of such support reinforced feelings of invisibility and questioning of their maternal role (30, 45). In situations where mothers did not feel their autonomy was respected, for example, when they were pressured to hold the baby against their will (43), this denial of their wishes intensified not only their suffering but also their difficulty in recognizing themselves as mothers in a grieving context. In contrast, strategies such as memory boxes and peer support mentioned in specialized services helped validate their experience, strengthening a continuous sense of motherhood (45). The way grief was named also influenced this identity reconstruction. Expressions such as *“rainbow baby”*, even when not explicitly explained, functioned as symbolic elements which connected the past loss to a future hope, allowing women to see their journey as part of a continuous process (30). Furthermore, by supporting other bereaved mothers, many re-signified their own pain, reaffirming their identity not only as women who experienced loss but also as those who offer support and understanding (45).

Despite the pain, some women were able to find new meaning in their motherhood, particularly through supporting other bereaved mothers. Maternal identity was then expanded and connected to the collective. Mutual support, as well as recognition between partners, also proved essential for constructing maternal identity, especially when the surrounding society failed to embrace these women’s pain (38). In another study, the bond with a surviving twin seemed to emerge from the day of the funeral, through words and caresses encouraged by caregivers; this was how she became a mother (36).

#### 4.3.2 Re-interpreting loss after birth: coping with grief and emotions in a subsequent pregnancy

The studies showed that pregnancy following loss is experienced as a unique and highly sensitive journey, marked by intense emotions and ongoing grief (41, 45). Experiencing motherhood after a perinatal bereavement brings with it the need to reinterpret the loss in light of the arrival of a new baby. This process requires women to make a continuous emotional effort to bond with the new child without erasing or denying the memory of the one who was lost (37, 39, 42).

This process is often accompanied by intense fears, insecurities, and anxiety. The fear of experiencing another loss leads many women to adopt an emotionally restrained posture during the subsequent pregnancy as a form of self-protection from previous trauma (32, 37). Emotional detachment during pregnancy appears, in some cases, as a survival strategy. Even as childbirth approaches, painful memories of the previous loss may resurface, reactivating grief and making it difficult to fully experience the new pregnancy (37). Despite the birth of a healthy baby, grief does not end, and it is common for feelings of fear and concern about the possibility of another loss to persist. Some women reported difficulty in forming an emotional bond during pregnancy, although, over time, many developed a positive relationship with their new child. The arrival of this baby also triggered a re-interpretation of the previous loss, influencing how mothers made sense of their experiences. In some cases, there was an attempt at symbolic substitution of the deceased child, evidenced by the choice of similar names or the reuse of clothes and items that had belonged to the baby who died (41).

The pursuit of a new pregnancy is sometimes described as an attempt to regain a sense of life (36). However, the expected joy of a new pregnancy is often replaced by constant emotional vigilance and feelings of guilt. These aspects make prenatal care a fundamental space for support and attentive listening. The literature emphasizes that sensitive and individualized care is essential during a subsequent pregnancy (41). Nevertheless, some women avoid activities such as prenatal classes, associating them with the pain of their earlier experience (31).

After birth, the baby may carry deep symbolic meaning. Many mothers report ambivalent feelings, revealing the emotional and symbolic impact the new child represents (41). Although there is an effort to bond with the baby who was born, there remains an understanding that this child does not replace the one who was lost. Symbolic language is a strategy used by some women to organize their experiences and socially communicate the complexity of this new beginning (30, 41). This process shows grief does not end with the birth of another child but continues to coexist with the life that begins anew. The first years of the new child’s life remain intertwined with the memory of the previous loss, demonstrating that a subsequent pregnancy demands a continuous and delicate emotional journey (36).

## 5 Analytic-synthesis and the meta-ethnographic theory

**Figure 2:**
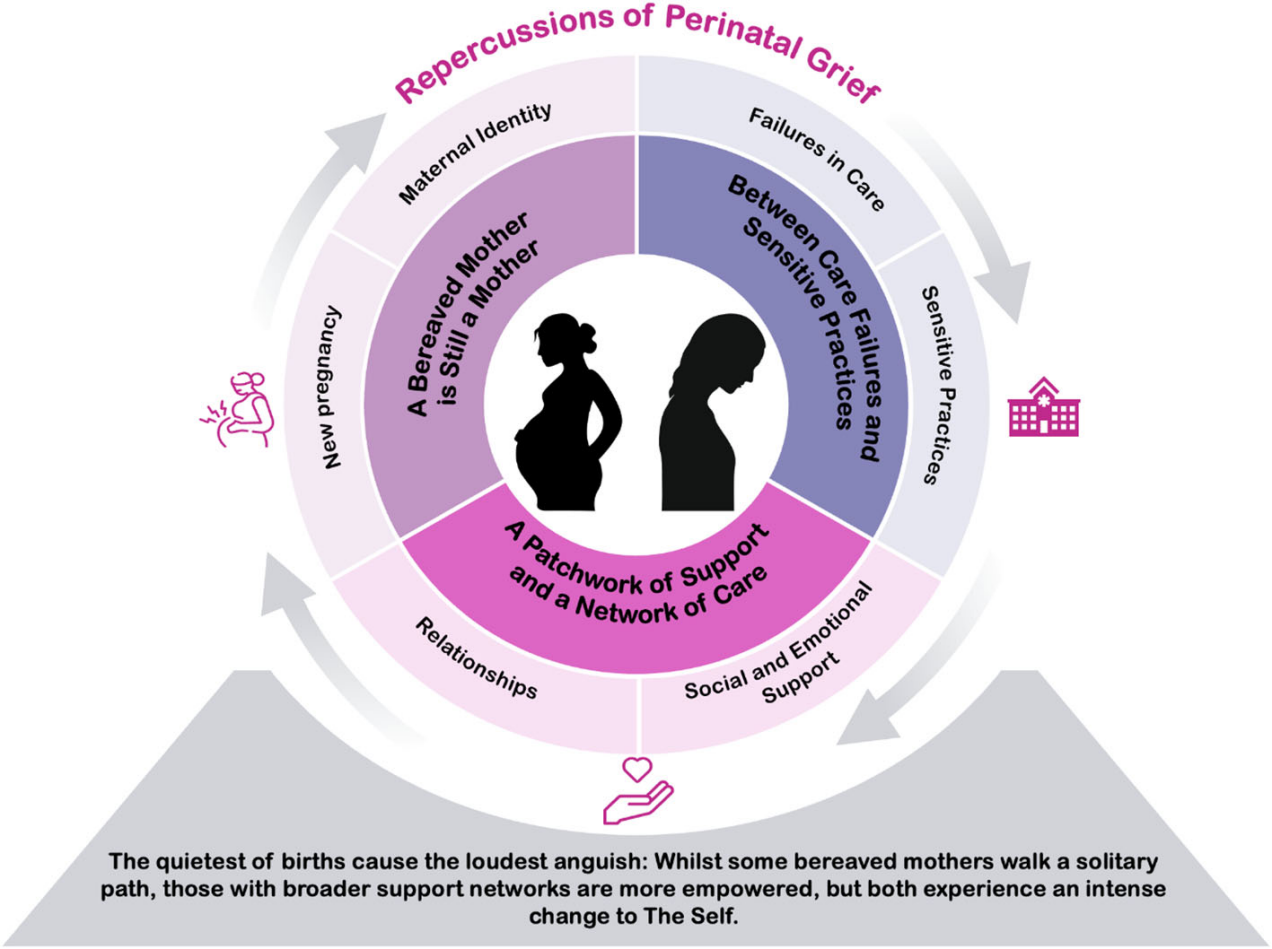
Illustration of the final theory.

Perinatal grief is deeply marked not only by the silence surrounding each loss-marked birth, but also by how it is acknowledged or silenced within institutional and social contexts. The more silent and invisible the perinatal bereavement is, the more intense the anguish experienced by mothers tends to be, as they find themselves isolated, guilt-ridden, and emotionally unsupported. The lack of recognition of the loss by healthcare professionals, family, and friends deepens the suffering and weakens maternal identity. On the other hand, women who have stronger, more empathetic support networks with attentive listening, validation of their pain, and longer-term consistent care become more empowered to reinterpret the loss and rebuild their experience of motherhood. The interpretative theory that synthesizes the findings of this review can be expressed as follows: *“The quietest of births cause the loudest anguish: Whilst some bereaved mothers walk a solitary path, those with broader support networks are more empowered, but both experience an intense change to The Self.”* (**Figure 2**).

## 6 Discussion

This meta-ethnography aimed to synthesize qualitative studies on the experience and long-term repercussions of perinatal grief in women who have lost their babies. The analytic-synthesis of findings revealed that perinatal grief constitutes a unique experience, deeply marked by emotional suffering, social silencing, and identity disruption, indicating this experience does not end with the loss event, but continues and transforms over time. Women face not only the pain of the loss itself but also emotional neglect, insensitive communication from healthcare professionals, and stigmatization within their social contexts. The lack of qualified support and the symbolic invalidation of motherhood after the loss generate feelings of loneliness, exclusion, and helplessness. On the other hand, experiences in which there was empathic care, continuity of support, and recognition of the pain experienced showed the potential to alleviate suffering and support the subjective reconstruction of motherhood. These results highlight that the way the loss is addressed by both healthcare services and social networks directly influences the grieving process and emotional trajectory of bereaved women. Based on these findings, the following theory was developed: *“The quietest of births cause the loudest anguish: Whilst some bereaved mothers walk a solitary path, those with broader support networks are more empowered, but both experience an intense change to The Self.”*.

The findings of this review reveal that gaps in perinatal bereavement care are systemic, ranging from the early identification of prenatal risk to the continuity of support in the postpartum period. Among the main issues identified are the lack of a welcoming environment for the emotional expression of parents, the scarcity of clear information, and insensitive communication from professionals unprepared to deal with situations of loss. These results align with the literature, which pointed to delays in communicating the death, the use of vague terminology, and even withholding information under the pretext of ‘protecting’ the mother (47). Similarly, the intensified suffering of women placed in wards alongside mothers and live newborns was highlighted, in settings marked by a lack of privacy and sensitivity (48). These factors heighten the emotional impact of the loss, hinder the grieving process, and reveal persistent gaps in professional training and conduct in the face of perinatal death.

These findings can be theoretically understood through the Dual Process Model of Coping with Bereavement (23). Within this framework, women’s experiences of perinatal grief can be interpreted as a continuous movement between confronting the emotional pain of loss expressed through sadness, guilt, and disrupted maternal identity and engaging in restorative actions that allow reconstruction of meaning and self-identity. The themes identified in this meta-ethnography resonate with this duality: while loss-oriented processes reflect the emotional intensity and identity rupture following perinatal death, restoration-oriented processes are evident in the rebuilding of motherhood and the search for validation through social and professional support networks. Thus, the integration of Stroebe and Schut’s model strengthens the theoretical interpretation of the synthesized findings, offering a conceptual framework to understand how bereaved mothers navigate between pain and adaptation over time (23).

While the Dual Process Model highlights the importance of restoration-oriented coping in mitigating grief, the effectiveness of such strategies is often constrained by systemic and institutional shortcomings. Even when institutional guidelines exist, their implementation is often inconsistent. As observed in the literature, few professionals report feeling prepared to offer adequate support, revealing the absence of specific protocols and the fragmentation of follow-up care (49). In-line with the findings, such weaknesses are exacerbated in contexts of social vulnerability, where neglect, lack of information, and barriers to accessing care compromise prenatal follow-up and lead to traumatic birth experiences (50). It is therefore essential that institutional protocols be applied in a flexible and context-sensitive manner, guided by the multidisciplinary team, so that they support rather than restrict individualized and compassionate care that addresses the emotional and psychological needs of bereaved mothers.

Although not all studies detailed aspects such as income, occupation, or access to health insurance, it was possible to observe both positive and negative experiences occurred across different socio-economic contexts. However, more favorable experiences were associated with the presence of specialized services with teams trained to offer sensitive care in the context of loss. In line with the findings, specialized services not only provided a protective, welcoming, and emotionally safe environment, but women also emphasized the value of continuity with the same professionals, which prevented the painful repetition of their stories and contributed to a greater sense of being supported (51). In contrast to reports of negligence, some studies highlighted compassionate practices such as active listening, emotional support, and relational care, particularly in institutions with well-prepared multi-disciplinary teams. These approaches were perceived as facilitators in the grief process and demonstrate the transformative impact of woman-centered care (52).

The quality of emotional support from professionals, family members, and partners emerges as a decisive factor in healthier coping with perinatal bereavement. Both the literature and the findings of this review indicate that empathic support contributes to validating the experience, strengthening bonds, and promoting resilience. Conversely, as studies have shown, its absence tends to intensify suffering and negatively affect mothers’ mental health (47). In addition, the research reinforces that support from close family members especially mothers, mothers-in-law, and siblings played a central role in the emotional care and restructuring of these women, even when that support was expressed in rigid or culturally mediated ways (47). The emotional consequences of loss, often marked by feelings of guilt, helplessness, and deep sadness, affect both family and social relationships, which may be strengthened or, conversely, weakened in the face of grief. Consistent with the literature, studies indicate these emotions are associated with anxiety, depression, chronic pain, and intense emotional reactions, frequently reported after perinatal bereavement (53, 54).

The impact of perinatal bereavement, however, goes beyond clinical and family settings. It is shaped by sociocultural constructions that silence or delegitimize this type of grief, making its symbolic processing more difficult. Other studies reinforce these findings by showing that families wish to name the baby, say goodbye, and preserve memories as a way to acknowledge the baby’s existence and validate the pain experienced. The denial of such rituals contributes to the social invalidation of grief. When these rituals are denied, the grief becomes socially disenfranchised. The absence of social recognition of motherhood and of the pain experienced intensifies feelings of exclusion and ambivalence, further weakening maternal identity (53). Women recognize themselves as mothers, but without external validation, they feel invisible, something that affects emotional bonds and deepens suffering. In-line with the results of this review, synthesis, and meta-ethnography, it was found that this identity tension can extend into subsequent pregnancies, often permeated by fear, guilt, and anxiety. In such cases, the bond with the new baby tends to be formed cautiously as a strategy of emotional self-protection (55).

Finally, this review revealed significant knowledge gaps regarding the effects of the recent pandemic on perinatal grief, particularly in its long-term dimension. Major changes in healthcare services, such as the cancellation of appointments and exams (56), and restrictions on contact with the baby after death, further intensified women’s suffering during this period (57, 58). The absence of farewell rituals and distancing from family and emotional support networks made the loss even more difficult to process (59). The pandemic not only intensified pre-existing vulnerabilities but also profoundly reshaped the experience of perinatal grief (60–62), with potentially long-lasting consequences.

## 7 Strengths, limitations, and future directions

The strengths of this review are related to the methodological choice of meta-ethnography, which enables a rich and theoretical understanding of lived experiences, allowing for a deep interpretative synthesis. The use of the CERQual tool (25) enhances the transparency and credibility of the findings. In addition, the critical analytic-synthesis of the studies allowed for the identification of important gaps in the literature, such as the lack of investigations into long-term perinatal grief in pandemic contexts. As for limitations, it is noted that not all included studies provided detailed information on socio-economic aspects, such as access to health insurance, family income, or urban/rural location. Moreover, differences in cultural contexts and theoretical frameworks among the studies posed some challenges to the direct comparison of findings. Furthermore, although the included studies exhibited some geographical diversity, most were conducted in high- or upper-middle-income settings, which may limit the comprehensiveness of the synthesis in an intercultural context, as well as the generalizability of our findings. These limitations may have influenced the interpretative synthesis, shaping which experiences were emphasized and how themes were constructed. This reflexive consideration highlights that our findings should be interpreted with caution, particularly in low- and middle-income contexts.

Furthermore, no studies were found that specifically addressed long-term grieving experiences (more than one year after the loss) during or after the pandemic. This gap underscores the urgency of future research to explore in depth the prolonged effects of the pandemic on perinatal grief, contributing to the improvement of public policies and care practices for bereaved families. The absence of studies addressing long-term experiences related to loss due to fetal malformation also represents a knowledge gap, highlighting the scarcity of research focused on perinatal grief associated with this type of loss. Future studies should also consider employing longitudinal qualitative designs to capture evolving grief experiences over time, triangulation with mixed methods to enhance the comprehensiveness and validity of findings, and multicultural research approaches to improve representativeness and generalizability. Moreover, even with the mothers being the focus of our study, fathers’ support and experience were little mentioned by the participants, raising opportunities for future studies on the grief of fathers. In this regard, interdisciplinary research that integrates perspectives from different disciplines can offer deeper insights into the complexity of parental grief and inform more comprehensive and responsive care strategies.

## 8 Conclusion

This systematic review, qualitative evidence synthesis, and meta-ethnography presents the experiences and long-term repercussions of perinatal grief in women who have suffered a perinatal bereavement. The analytic-synthesis revealed that perinatal grief is a complex and prolonged experience, marked by intense emotional suffering, social silencing, and disruptions in maternal identity. The lack of adequate support and the insensitive communication from healthcare professionals contribute to the isolation and exclusion of these women. On the other hand, empathic care, acknowledgment of their pain, and continuity of support emerge as essential factors for alleviating suffering and supporting the subjective reconstruction of motherhood over the life course from lost to subsequent pregnancy. Bereaved mothers follow different trajectories, with those who have broader and more supportive networks coping better with their loss. Thus, the functioning of social and institutional support systems around the woman directly influences her grieving process and the personal transformation that follows the loss. These findings underscore the need for clinical practice to prioritize individualized, compassionate, and continuous care; for education to include structured training in grief support and communication; for research to further investigate culturally specific experiences and long-term outcomes among bereaved women.
