# Supplementary material for: Experiences and long-term repercussions of perinatal grief in women after perinatal bereavement: a meta-ethnography
Source: Front Psychiatry. 2025 Dec 17;16:1661483. doi: 10.3389/fpsyt.2025.1661483 (PMC12754219; doi:10.3389/fpsyt.2025.1661483)
Supplement: Supplementary file 1 [file Table1.docx]

Supplementary Material

**Supplementary Table 1:** PubMed search strategy based on the SPIDER mnemonic.

| **SPIDER** | **#** | **Search Terms** |
| --- | --- | --- |
| **S**ample | #1 | ((((("bereaved mother" [tiab]) OR ("Women"[Mesh] OR "Mothers"[Majr])) |
| **P**henomenon  of **I**nterest | #2 | (("Perinatal Death"[Mesh] OR "Perinatal Mortality"[Mesh] OR "Fetal Death"[Mesh] OR "Stillbirth"[Mesh] OR "Abortion, Spontaneous"[Mesh] OR "Grief"[Mesh] OR "Bereavement"[Mesh] OR "perinatal grief" [tiab] OR "perinatal bereavement" [tiab] OR "perinatal loss" [tiab]) OR ("Infant, Extremely Premature"[Mesh])) OR ("Premature Birth"[Mesh])) OR ("Infant, Premature"[Mesh])) |
| **D**esign | #3 | ((((("semi-structured"[TIAB] OR semistructured[TIAB] OR unstructured[TIAB] OR informal[TIAB] OR "in-depth"[TIAB] OR indepth[TIAB] OR "face-to-face"[TIAB] OR structured[TIAB] OR guide[TIAB] OR guides[TIAB]) AND (interview*[TIAB] OR discussion*[TIAB] OR questionnaire*[TIAB])) OR ("focus group"[TIAB] OR "focus groups"[TIAB] OR qualitative[TIAB] OR ethnograph*[TIAB] OR fieldwork[TIAB] OR "field work"[TIAB] OR "key informant"[TIAB])) OR "interviews as topic"[Mesh] OR "focus groups"[Mesh] OR narration[Mesh] OR qualitative research[Mesh] OR "personal narratives as topic"[Mesh]) OR (((((((("Attitude"[Mesh]) OR "Optimism"[Mesh]) OR "Emotions"[Mesh]) OR "Comprehension"[Mesh]) OR ("view*" [tiab])) OR ("experienc*" [tiab])) OR ("opinion*" [tiab])) OR ("attitude*" [tiab]))) |
| **E**valuation | #4 | ((((((("Risk Assessment"[Mesh]) OR ("Health Education"[Mesh])) OR ("Family Health"[Mesh])) OR ("Quality of Life"[Mesh])) OR ("Life Change Events"[Mesh])) OR ("Emotions"[Mesh])) OR ((Experience) OR (Experiences) OR (Sense) OR (Senses) OR (Meaning) OR (Meanings) OR (Psychologic) OR (Mental Health) OR (Struggling) OR (Impacts) OR (Influences) OR (Life Change Event) OR (Life Experience) OR (Experience, Life) OR (live experience) OR Adaptation, Psychological[Mesh] OR (Adaptation, Psychologic) OR (Psychologic Adaptation) OR (Psychological Adaptation) OR (Adjustment) OR (Coping Behavior) OR (Behavior, Coping) OR (Behaviors, Coping) OR (Coping Behaviors) OR (Coping Skills) OR (Coping Skill) OR (Skill, Coping) OR (Skills, Coping) OR (Coping Strategies) OR (Coping Strategy)OR (Strategies, Coping) OR (Strategy, Coping) OR (Behavior, Adaptive) OR (Adaptive Behavior) OR (Adaptive Behaviors) OR (Behaviors, Adaptive) OR (Perspectives) OR (Feelings)))) |
| **R**esearch Type | #5 | ((("Research, Qualitative"[Text Word] OR "Qualitative studies"[Text Word] OR "Research, Empirical"[Text Word] OR "qualitative methods"[Text Word] OR qualitative[Text Word]) OR ("Qualitative Research"[Mesh] OR "Empirical Research"[Mesh]))) |
| **SPIDER** | #6 | #1 AND #2 AND AND #3 AND #4 AND #5 |

**Supplementary Table 2:** Quality appraisal according to the JBI Critical Appraisal Checklist for Qualitative Research.

| **First author and**  **year**  **JBI Checklist**  **questions** | **Borges 2024^(34)^** | **Rossen, 2023^(38)^** | **Teodózio, 2022^(41)^** | **Wheeler, 2022^(30)^** | **Lopes, 2021^(42)^** | **Testoni, 2020^(29)^** | **Devincenzi, 2019^(35)^** | **Due, 2018^(44)^** | **Jordan, 2018^(40)^** | **Meredith, 2017^(45)^** | **Golan, 2016^(39)^** | **Üstündağ-Budak, 2015^(37)^** | **Gagnon, 2013^(46)^** | **Vasilescu, 2013^(36)^** | **Yamazaki, 2010^(33)^** | **Gerber-Epstein, 2009^(31)^** | **St John, 2006^(32)^** | **Lundqvist, 2002^(42)^** |
| --- | --- | --- | --- | --- | --- | --- | --- | --- | --- | --- | --- | --- | --- | --- | --- | --- | --- | --- |
| **1. Is there congruity between the stated philosophical perspective and the research methodology?** | **Y** | **Y** | **U** | **U** | **U** | **Y** | **N** | **Y** | **N** | **Y** | **Y** | **U** | **Y** | **U** | **Y** | **U** | **Y** | **U** |
| **2. Is there congruity between the research methodology and the research question or objectives?** | **Y** | **Y** | **Y** | **Y** | **Y** | **Y** | **U** | **Y** | **Y** | **Y** | **Y** | **U** | **Y** | **Y** | **Y** | **U** | **Y** | **Y** |
| **3. Is there congruity between the research methodology and the methods used to collect data?** | **Y** | **Y** | **Y** | **Y** | **Y** | **Y** | **Y** | **Y** | **Y** | **Y** | **Y** | **U** | **Y** | **Y** | **Y** | **Y** | **Y** | **Y** |
| **4. Is there congruity between the research methodology and the representation and analysis of data?** | **Y** | **Y** | **U** | **Y** | **Y** | **Y** | **U** | **Y** | **Y** | **U** | **Y** | **U** | **Y** | **Y** | **Y** | **Y** | **Y** | **Y** |
| **5. Is there congruity between the research methodology and the interpretation of results?** | **Y** | **Y** | **Y** | **Y** | **Y** | **Y** | **Y** | **Y** | **Y** | **Y** | **Y** | **Y** | **Y** | **Y** | **Y** | **U** | **Y** | **Y** |
| **6. Is there a statement locating the researcher culturally or theoretically?** | **N** | **Y** | **N** | **N** | **N** | **N** | **N** | **Y** | **N** | **Y** | **N** | **Y** | **N** | **N** | **N** | **N** | **N** | **N** |
| **7. Is the influence of the researcher on the research and vice- versa addressed?** | **N** | **Y** | **N** | **N** | **N** | **U** | **N** | **U** | **U** | **Y** | **U** | **Y** | **U** | **Y** | **U** | **U** | **N** | **U** |
| **8. Are participants and their voices adequately represented?** | **Y** | **Y** | **Y** | **Y** | **Y** | **Y** | **Y** | **Y** | **Y** | **Y** | **Y** | **Y** | **Y** | **Y** | **Y** | **Y** | **Y** | **Y** |
| **9. Is the research ethical according to current criteria or for recent studies and is there evidence of ethical approval by an appropriate body?** | **Y** | **Y** | **Y** | **Y** | **Y** | **Y** | **Y** | **Y** | **Y** | **Y** | **U** | **Y** | **U** | **Y** | **Y** | **Y** | **Y** | **Y** |
| **10. Do the conclusions drawn in the research report flow from the analysis or interpretation of the data?** | **Y** | **Y** | **Y** | **Y** | **Y** | **Y** | **Y** | **Y** | **Y** | **Y** | **Y** | **Y** | **Y** | **Y** | **Y** | **Y** | **Y** | **Y** |
| ***N/B.*** Y = Yes \| N = No \| U = Unclear | | | | | | | | | | | | | | | | | | |

# 2. Supplementary Table 3: Evidence Profile according to GRADE-CERQUAL.

| **#** | **Summarised review finding** | **Methodological limitations** | **Coherence** | **Adequacy** | **Relevance** | **GRADE-CERQual assessment of confidence** | **References** | |
| --- | --- | --- | --- | --- | --- | --- | --- | --- |
| **1.**  **BETWEEN CARE FAILURES AND SENSITIVE PRACTICES** | | | | | | | |  |
| **1** | **Impact of health service deficiencies on perinatal loss experience.** | Moderate concerns  **Explanation:** Moderate concerns regarding methodological limitations because two out of three studies did not address the cultural or theoretical positioning of the researcher or reflexivity. These aspects were either absent or not clearly described, which limits the transparency and depth of the analytical process. | Minor concerns  **Explanation:** Minor concerns regarding coherence because although there appear to be conflicting findings regarding the quality of prenatal care associated with perinatal loss, these differences are explained by the distinct contexts of the included studies. Studies from specialized clinics report adequate prenatal care, whereas research conducted in vulnerable and resource-limited settings points to deficiencies in care. This contextual variation is plausible and expected, reflecting real differences in healthcare access and quality across settings. | Minor concerns  **Explanation:** Minor concerns regarding adequacy because of the few studies supporting this finding. | Moderate concerns  **Explanation:** Moderate concerns regarding relevance because the included studies represent different contexts: one study was conducted in a specialized service within a developed country, while two others were from public health settings in developing countries. Although all studies involve women who experienced perinatal loss, the differences in healthcare systems and resource availability mean that the findings may not be equally applicable across all settings. | Moderate confidence  **Explanation:** Moderate concerns regarding methodological limitations, Minor concerns regarding coherence, Minor concerns regarding adequacy, and Moderate concerns regarding relevance | (34-35,45) | |
| **2** | **Failures in care and labor management had significant impacts.** | Moderate concerns  **Explanation:** Moderate concerns regarding methodological limitations because three out of four studies did not address the cultural or theoretical positioning of the researcher or reflexivity. These aspects were either absent or not clearly described, which limits the transparency and depth of the analytical process. | Minor concerns  **Explanation:** Minor concerns regarding coherence because as the review finding incorporates and explains the variation in primary data including both positive evaluations and reported failures in psychological support. The apparent contradictions are meaningfully integrated into the synthesis, reflecting the complexity of participants’ experiences across different contexts. | Moderate concerns  **Explanation:** Moderate concerns regarding adequacy because of the few studies supporting this finding. | Minor concerns  **Explanation:** Minor concerns regarding relevance because The contexts of care vary between public, private, and specialized clinical services, but all are relevant to the research. | Moderate confidence  **Explanation:** Moderate concerns regarding methodological limitations, Minor concerns regarding coherence, Moderate concerns regarding adequacy, and Minor concerns regarding relevance | (35-36,43-44) | |
|  |  |  |  |  |  |  |  | |
| **3** | **Absence of postpartum follow-up led to feelings of abandonment and isolation.** | Moderate concerns  **Explanation:** Moderate concerns regarding methodological limitations because There are moderate concerns about methodological limitations, as three studies did not address aspects such as the cultural or theoretical positioning of the researcher, and four studies did not address reflexivity or did so unclearly. One study did not clearly describe the congruence between the research methodology and the research question or objectives, as well as between the methodology and the representation and analysis of the data. | No/Very minor concerns | No/Very minor concerna | No/Very minor concerns | Moderate confidence  **Explanation:** Moderate concerns regarding methodological limitations, No/Very minor concerns regarding coherence, No/Very minor concerns regarding adequacy, and No/Very minor concerns regarding relevance | (32,34-35,44) | |
| **4** | **Empathetic care and institutional support more prevalent in specialized services.** | Moderate concerns  **Explanation:** Moderate concerns regarding methodological limitations because there are moderate concerns about methodological limitations. Reflexivity was not addressed or was unclear in two of the studies, and one study did not clearly describe the congruence between the research methodology and data analysis. | Minor concerns  **Explanation:**  Minor concerns regarding coherence because although predominant in specialized services, other services also provided empathetic care. | Minor concerns  **Explanation:** Minor concerns regarding adequacy because Minor concerns regarding adequacy because of the few studies supporting this finding. | Minor concerns  **Explanation:** Minor concerns regarding relevance because The studies were conducted in geographically distinct contexts (Australia and Brazil), encompassing both public and private healthcare systems." | Moderate confidence  **Explanation:** Moderate concerns regarding methodological limitations, No/Very minor concerns regarding coherence, Minor concerns regarding adequacy, and Minor concerns regarding relevance | (34,44-45) | |
|  |  |  |  |  |  |  |  | |
| **5** | **Psychotherapy groups were identified as important sources of emotional support.** | Moderate concerns  **Explanation:** Moderate concerns regarding methodological limitations because as all three studies lacked a clear statement regarding the cultural or theoretical positioning of the researcher. Additionally, two of the three studies did not adequately address reflexivity or the influence of the researcher on the research process. | Minor concerns  **Explanation:** Minor concerns regarding coherence because Two studies provide rich and consistent evidence: one highlights the positive emotional impact of psychotherapy groups, while the other describes ongoing psychological support during and after hospitalization as crucial for emotional processing. The third study does not directly address psychological support but does not contradict the finding. | Minor concerns  **Explanation:** Minor concerns regarding adequacy because because of the few studies supporting this finding. | Minor concerns  **Explanation:** Minor concerns regarding relevance because All included studies were conducted in high-income countries, which may limit the transferability of the findings to low- and middle-income settings. | Moderate confidence  **Explanation:** Moderate concerns regarding methodological limitations, Minor concerns regarding coherence, Minor concerns regarding adequacy, and Minor concerns regarding relevance | (29,36,46) | |
| **6** | **Symbolic and personalized gestures had a meaningful emotional impact on bereaved women.** | Moderate concerns  **Explanation:** Moderate concerns regarding methodological limitations because There were moderate concerns regarding one study that did not clearly present the philosophical perspective underpinning the research methodology or the cultural positioning of the researcher. Another study did not demonstrate congruence between the chosen methodology and the representation and analysis of the data, and a third study did not address reflexivity. | No/Very minor concerns | Minor concerns  **Explanation:** Minor concerns regarding adequacy because Minor concerns regarding adequacy because of the small number of studies reporting this finding. | Moderate concerns  **Explanation:** Moderate concerns regarding relevance because Although the number of studies is relatively limited, the depth and consistency of the data are sufficient to support the finding. A broader representation of diverse cultural settings and different types of perinatal loss would further strengthen adequacy. | Moderate confidence  **Explanation:** Moderate concerns regarding methodological limitations, No/Very minor concerns regarding coherence, Minor concerns regarding adequacy, and Moderate concerns regarding relevance | (30,44-45) | |
|  |  |  |  |  |  |  |  | |
| **2. A PATCHWORK OF SUPPORT AND A NETWORK OF CARE** | | | | | | | |  |
| **7** | **Internal Emotional Struggles Amid the Invisibility of Perinatal Grief** | Moderate concerns  **Explanation:** Moderate concerns regarding methodological limitations because several studies did not clearly state the philosophical perspective underpinning the methodology or failed to culturally or theoretically locate the researcher. Specifically, five studies lacked a statement locating the researcher, while one study showed multiple unclear items regarding congruence between methodology and data collection, analysis, and interpretation. | No/Very minor concerns | Minor concerns  **Explanation:** Minor concerns regarding adequacy because some studies focus more on the emotional dimension, others on social relationships, but all are complementary to the finding. | No/Very minor concerns  **Explanation:**  Minor concerns regarding relevance because few studies focuses more on the period immediately following the loss, which may slightly limit their applicability to long-term experiences. | Moderate confidence  **Explanation:** Moderate concerns regarding methodological limitations, No/Very minor concerns regarding coherence, Minor concerns regarding adequacy, and No/Very minor concerns regarding relevance | (29,32-34,36-38,40-45) | |
| **8** | **Social Invalidation and the Intensification of Perinatal Grief** | Serious concerns  **Explanation:** Serious concerns regarding methodological limitations because several studies did not clearly state the philosophical perspective underpinning the research or failed to culturally or theoretically locate the researcher. Specifically, eight studies did not provide a statement locating the researcher. Additionally, two studies did not clearly address ethical approval. Reflexivity was either not addressed or unclear in most studies. | No/Very minor concerns | Minor concerns  **Explanation:** Minor concerns regarding adequacy because of a slight limitation in the temporal focus in some studies. | Minor concerns  **Explanation:** Minor concerns regarding relevance because a small number of studies focus more on the immediate aftermath of loss rather than exclusively on long-term repercussions, which may slightly limit the scope related to long-term experiences. | Moderate confidence  **Explanation:** Serious concerns regarding methodological limitations, No/Very minor concerns regarding coherence, Minor concerns regarding adequacy, and Minor concerns regarding relevance | (29,32-34,36,38-40,45-46) | |
|  |  |  |  |  |  |  |  | |
| **9** | **The Central Role of Partner Support in Coping with Grief** | Moderate concerns  **Explanation:** Moderate concerns regarding methodological limitations because all three studies did not provide a statement locating the researcher culturally or theoretically, and reflexivity was either not addressed or unclear. Additionally, one study showed several unclear responses related to congruence between methodology, data collection, data analysis, and interpretation. | No/Very minor concerns | Minor concerns  **Explanation:** Minor concerns regarding adequacy because of the small number of studies supporting this finding. | Minor concerns  **Explanation:** Minor concerns regarding relevance because there are studies from different continents, but all are from developed countries. | Moderate confidence  **Explanation:** Moderate concerns regarding methodological limitations, No/Very minor concerns regarding coherence, Minor concerns regarding adequacy, and Minor concerns regarding relevance | (29,33,37) | |
| **10** | **Ruptures and reconnections in social bonds in the face of loss** | Moderate concerns  **Explanation:** Moderate concerns regarding methodological limitations because three studies did not provide a statement locating the researcher culturally or theoretically, and reflexivity was not addressed. One study showed multiple unclear responses related to methodological congruence, including data collection, analysis, and interpretation. | Minor concerns  **Explanation:** Minor concerns regarding coherence because fewer studies address the reconnection dimension, the evidence is limited; however, it is explicit and complements the rupture theme by illustrating an important counterpoint of emotional reconnection and support. | Moderate concerns  **Explanation:** Moderate concerns regarding adequacy because in this case, the limited number of studies addressing reconnection indicates a moderate adequacy for this aspect of the finding. That is, most studies addressed the ruptures, and few studies focused on the reconnection. | No/Very minor concerns | Moderate confidence  **Explanation:** Moderate concerns regarding methodological limitations, Minor concerns regarding coherence, Moderate concerns regarding adequacy, and No/Very minor concerns regarding relevance | (29-31,34,42) | |
|  |  |  |  |  |  |  |  | |
| **3. A BEREAVED MOTHER IS STILL A MOTHER** | | | | | | | |  |
| 11 | Feeling of exclusion from the maternal experience due to the absence of the baby and physical/social markers of motherhood | Moderate concerns  **Explanation:** Moderate concerns regarding methodological limitations because six studies did not provide a statement locating the researcher culturally or theoretically, and reflexivity was either unclear or not addressed in five studies. In addition, one study did not clearly report the philosophical perspective guiding the study. | No/Very minor concerns | No/Very minor concerns | No/Very minor concerns | High confidence  **Explanation:** Moderate concerns regarding methodological limitations, No/Very minor concerns regarding coherence, No/Very minor concerns regarding adequacy, and No/Very minor concerns regarding relevance | (30-32,39-40,42) | |
| 12 | Role of emotional support in perinatal grief and maternal identity reconstruction. | Minor concerns  **Explanation:** Moderate concerns regarding methodological limitations because three of the studies, and one study did not provide a statement locating the researcher culturally or theoretically, and reflexivity was not addressed or was unclear in the same studies. Additionally, one study did not clearly present the philosophical perspective underpinning the research. | No/Very minor concerns | Minor concerns  **Explanation:** Minor concerns regarding adequacy because The data provide detailed descriptions of the role of emotional support in both adapting to perinatal grief and rebuilding maternal identity. However, three studies offer more concise contributions or are focused on only one of the two aspects of the finding. | No/Very minor concerns | Moderate confidence  **Explanation:** Minor concerns regarding methodological limitations, No/Very minor concerns regarding coherence, Minor concerns regarding adequacy, and No/Very minor concerns regarding relevance | (30,42-43,45) | |
| 13 | Finding meaning in motherhood through connection with other bereaved mothers | Minor concerns  **Explanation:** Minor concerns regarding methodological limitations because Minor concerns regarding methodological limitations. Two studies did not provide a statement locating the researcher culturally or theoretically, and reflexivity was not addressed in these two studies. | No/Very minor concerns | Minor concerns  **Explanation:** Minor concerns regarding adequacy because of the few studies supporting this finding. | Minor concerns  **Explanation:** Minor concerns regarding relevance because some studies place more emphasis on emotional growth, symbolic rituals, or individual coping than on explicit mutual support among bereaved women, which is the core of this finding. | Moderate confidence  **Explanation:** Minor concerns regarding methodological limitations, No/Very minor concerns regarding coherence, Minor concerns regarding adequacy, and Minor concerns regarding relevance | (36,38,45) | |
| 14 | Emotional self-protection during a new pregnancy after loss | Moderate concerns  **Explanation:** Moderate concerns regarding methodological limitations because most of the studies did not provide a statement locating the researcher culturally or theoretically, and reflexivity was either not addressed or unclear. | No/Very minor concerns | Minor concerns  **Explanation:** Minor concerns regarding adequacy because related to the depth of data in one of the studies, but without significantly compromising the overall interpretation of the finding. | No/ very Minor | Moderate confidence  **Explanation:** Moderate concerns regarding methodological limitations, No/Very minor concerns regarding coherence, Minor concerns regarding adequacy, and Minor concerns regarding relevance | (31-32,36,37,41) | |
| 15 | The baby’s symbolic and ambivalent meanings in the maternal narrative following loss | Minor concerns  **Explanation:** Minor concerns regarding methodological limitations because all three studies did not provide a statement locating the researcher culturally or theoretically. Additionally, reflexivity was not addressed or was unclear in two studies. | No/Very minor concerns | Minor concerns  **Explanation:** Minor concerns regarding adequacy because of the few studies supporting this finding. | No/Very minor concerns | Moderate confidence  **Explanation:** Minor concerns regarding methodological limitations, No/Very minor concerns regarding coherence, Minor concerns regarding adequacy, and No/Very minor concerns regarding relevance | (30,36,41) | |
